# Fascia’s role in the mind-body continuum: a novel target for integrative treatments in psychiatry

**DOI:** 10.3389/fpsyt.2026.1687288

**Published:** 2026-04-30

**Authors:** S Mudasser Shah, Muhammad Jahangir, Ghada Saleh Alhudaithi, Chand Taneja, Fatimah Sayer Alharbi, Xiuyun Lin

**Affiliations:** 1 Institute of Developmental Psychology, Beijing Normal University, Beijing, China; 2 Affiliated Mental Health Center & Hangzhou Seventh Peoples Hospital, Zhejiang University School of Medicine, Hangzhou, Zhejiang, China; 3 Self-Development Skills Department of common First Year Deanship, King Saud University, Riyadh, Saudi Arabia; 4 Department of Neuropsychology Program, Queen Alexandra Center for Children’s Health, Victoria, BC, Canada; 5 Department of Psychology and Division of Medical Sciences, University of Victoria, Victoria, BC, Canada; 6 Department of Health Sciences, College of Health and Rehabilitation Sciences, Princess Nourah Bint Abdulrahman University, Riyadh, Saudi Arabia; 7 Beijing Key Laboratory of Applied Experimental Psychology, Faculty of Psychology, Beijing Normal University, Beijing, China

**Keywords:** chronic stress, fascia, holistic therapy, mechanotransduction, myofascial pain, psychiatric disorders

## Abstract

The fascia, a pervasive connective tissue network, has traditionally been studied for its structural and biomechanical roles in the human body. However, emerging evidence suggests that fascia may also play a significant role in the pathophysiology of psychiatric disorders. This review article explores the potential links between fascial dysfunction and mental health conditions such as depression and anxiety. Fascia’s involvement in mechanotransduction, proprioception, and nociception positions it as a dynamic biological interface between peripheral tissues and the central nervous system. Through interoceptive signaling, the fascial system continuously relays the body’s internal physiological state to the brain, offering a plausible framework for understanding how somatic tension may correlate with emotional states. Integrating recent neurobiological frameworks, we propose that fascial afferents are primary contributors to higher-order body representation. Chronic stress induces fascial stiffness and inflammation, potentially exacerbating stress-related psychiatric conditions, while the comorbidity of myofascial pain syndromes with psychiatric disorders further highlights this interconnection. Although preliminary evidence suggests that fascial-targeted therapies including myofascial release, yoga, and meditation may hold therapeutic potential, their efficacy in psychiatric treatment remains hypothetical and requires validation through rigorous clinical trials. The evidence is still in infancy, integration of fascial health into psychiatric research and treatment offers a promising avenue for holistic and multidisciplinary approaches to mental health care. This article underscores the need for further research to elucidate the mechanisms underlying the fascia-psychiatry connection and to explore the clinical implications of fascial therapies in psychiatric practice.

## Introduction

The term “fascia” has traditionally been used in gross anatomy to refer to various undifferentiated mesenchymal tissues that either envelop organs and tissues or act as packing material between them (1–3). Over the years, its definition has evolved. Initially, it referred to structures such as a membranous tendon (4), a membranous part (5), or a robust aponeurotic band (6). Other interpretations included dense fibrous tissue surrounding internal organs (7), layers of connective tissue that could be either superficial or deep (8), and various types of connective tissue (9). The term even expanded to represent a global connective tissue system (10) and later to describe any dissectible mass of connective tissue (11). Recently, fascia has been described as a continuous, three-dimensional matrix that spans the entire body. It plays a crucial role in structural support and includes all collagen-based soft tissues responsible for forming and sustaining the extracellular matrix (12). This broader definition now includes tendons, ligaments, bursae, and the connective tissues surrounding muscles (such as the endomysium, perimysium, and epimysium) (13, 14). Fascia is a sheath of stringy connective tissue that surrounds every part of the body. It provides support to muscles, tendons, ligaments, tissues, organs, nerves, joints and bones.

In Western medicine, the term Fascia, and its four different types, which include connective tissues, endothelial, muscle and nerve tissues, have been overlooked and poorly understood in history. This has often been attributed to ongoing debates about its nomenclature, which may have distracted from clinically relevant fascia research (15). In the past, cellular membranes were considered passive structures, but it is now widely acknowledged that they play active, dynamic roles critical to cellular function. Previously thought to be inert, fascia has since been found to contain membrane-like receptors and exhibit functions akin to cellular membranes within the tissues it surrounds and supports (16), Current findings suggested the protective, supportive role of fascia, which surrounds various tissues in various systems of the body i.e., autonomic nervous systems, musculoskeletal, and endocrine systems. Numerous findings have highlighted the essential role in surrounding, supporting, and protecting not only in every nerve, but also in muscles, blood vessel, and other organs of the body (13, 17), Remarkably, fascia is estimated to contain over 250 million nerve endings (17), with sensory neurons outnumbering motor neurons by a ratio of 9:1 in certain regions (18). This high density of mechanoreceptors and free nerve endings positions the fascial system as a primary substrate for interoception; the process by which the nervous system senses and integrates signals regarding the internal state of the body (19). Within the scope of psychiatry, this interoceptive input is critical; fascial afferents do not merely relay mechanical data, but actively inform the central nervous system’s affective and emotional processing. Consequently, alterations in fascial tension may distort the brain’s internal representation of the body, potentially contributing to the emotional dysregulation observed in various psychiatric disorders (20). The fascial system is increasingly recognized as a primary sensory organ, playing a vital role in proprioception and interoception (21). It is characterized by a significantly higher concentration of sensory receptors compared to muscle tissue, with some estimates suggesting a sensory innervation up to six times greater than that of the musculoskeletal system (17). However, it is important to distinguish between total sensory volume and histological density. While the fascial network is ubiquitous, direct immunohistochemical comparisons have shown that the nerve fiber density within the superficial fascia is lower than that of the highly sensitive dermis (17, 22, 23).

**Figure 1:**
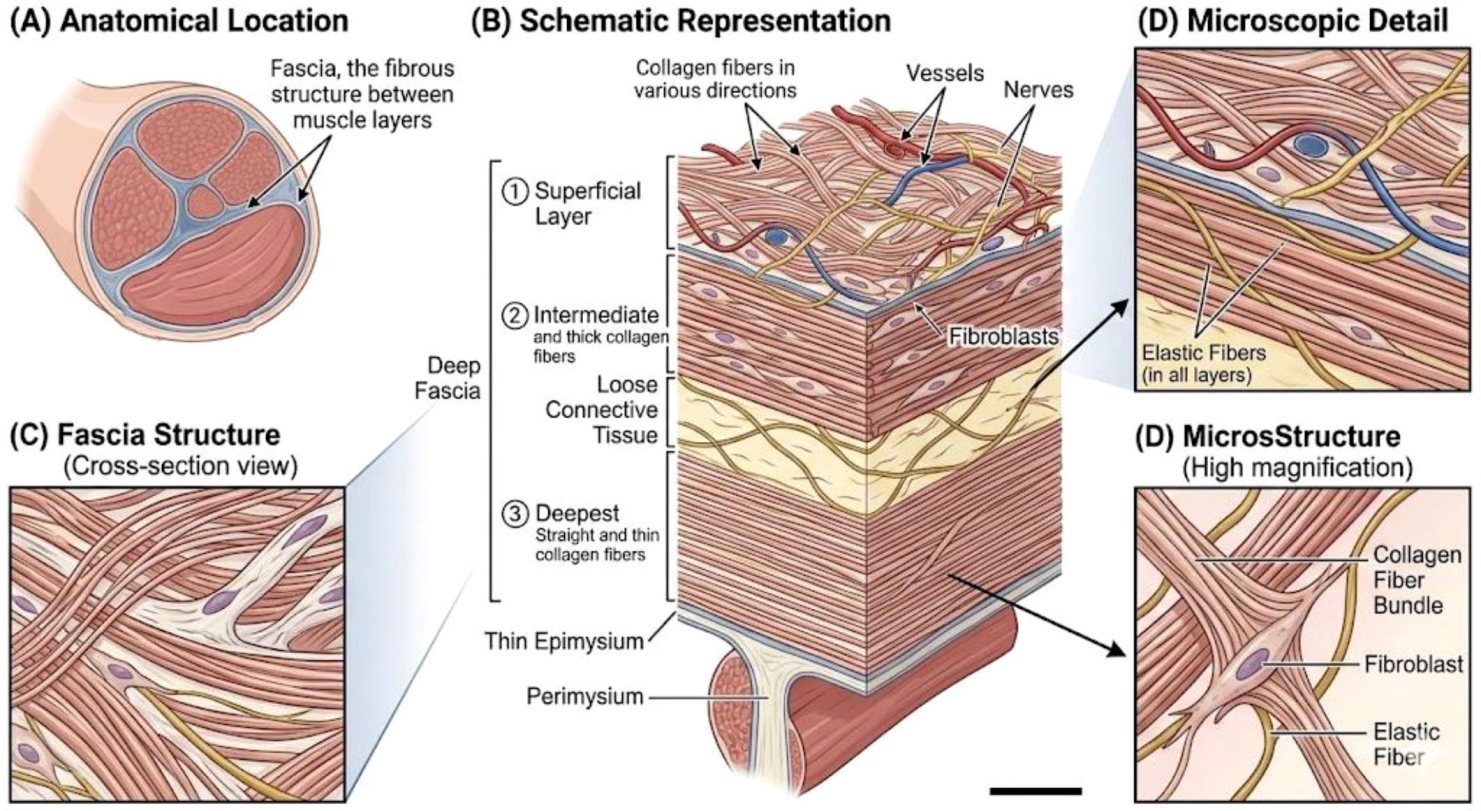
**(A)** Fascia, the fibrous structure between muscle layers, **(B)** schematic representation of the deep fascia, which is divided into three layers: the first superficial layer, containing collagen fibers extending in various directions with abundant vessels and nerves; the second intermediate layer, composed of single straight and thick collagen fibers with fibroblasts; and the third deepest layer, composed of straight and thin collagen fibers. A thin epimysium exists underneath the deep fascia and is connected to the perimysium. Loose connective tissue is identified between the second and third layers. Elastic fibers are found in all layers **(C, D)** Microscopic illustration of fascia’s structure.

Connective tissue, particularly fascial tissue, takes on various forms in the body. One form that can be found beneath the skin is loose connective tissue, while other is deep-connective tissue that can be found in various structures like epimysium, perimysium, and endomysium of muscles. It also includes cartilage, tendons, ligaments, the protective sheaths around nerves and blood vessels, and membranes like the periosteum, pericardium, and the visceral serous membrane. Historically, fascia was often overlooked during anatomical dissections focused on muscles, organs, neurovascular bundles, and bones. However, recent research has shed light on fascia’s emergence from obscurity, revealing it as one of the most functionally diverse components of the human body (24). As shown in **Figure 1**.

## Classification of fascia

Fascia can be classified as superficial, deep, visceral, or parietal and further classified according to anatomical location (25).

### 2.1 Superficial fascia

Superficial fascia consists of loose connective tissue located beneath the skin, containing a network of collagen fibers and some elastin fibers. This layer is absent in certain areas, such as the soles of the feet, palms of the hands. The tissue is typically thicker in size in some parts like trunk as compare to limbs, and gradually become thinner when it extends to the extremities. The superficial fascia often contains fat within its mesh. The superficial musculoaponeurotic system (SMAS) is a fibro-adipose-muscular network that connects facial muscles to the skin, enabling expression and fold formation. First described by Mitz and Peyronie in 1976 (26), it separates facial fat into layers and is essential for understanding aging and performing face-lift surgeries. Its role in pain or psychiatric symptoms, however, is not addressed in the available literature. The superficial fascia frequently integrates muscle fibers to form specialized functional units, such as the platysma in the cervical region, the external anal sphincter, and the dartos fascia within the scrotum. In the abdominal wall, this layer is specifically characterized as Scarpa’s fascia (14, 27, 28). Far from being a passive structural filler, the superficial fascia is essential for maintaining somatic morphology, ensuring vascular patency, and providing mechanical cushioning for underlying tissues. Crucially, it facilitates multidirectional gliding between the dermis and the deep musculature. Therapeutic interventions, including manual therapy, thermotherapy, and targeted stretching, are hypothesized to modulate the mechanoreceptors within this tissue to alleviate myofascial pain and reduce localized inflammation (29). Anatomically, the superficial fascia functions as a fibroelastic interface separating the superficial adipose tissue (SAT) from the deep adipose tissue (DAT). The SAT layer is physiologically active, contributing to metabolic homeostasis including glucose regulation as well as thermoregulation and immune signaling (30). Within this architecture, the superficial fascia acts as a conduit for hormonal transport and a protective sheath for neurovascular bundles (31). The inherent viscoelasticity of this fascial layer provides critical shock absorption, mitigating mechanical stress during movement (32). Finally, the structural integrity of the integumentary system is maintained by the retinacula cutis, collagenous septa, that anchor the dermis to the underlying fascial planes, ensuring structural stability while preserving the necessary mobility of the skin (33).

### 2.2 Deep fascia

Deep fascia is a fibrous connective tissue membrane that surrounds different parts of the body like muscles, nerves and blood vessels. Unlike superficial fascia, it does not contain fat and is typically more fibrous in texture. Deep fascia is rich in hyaluronan and is often highly vascularized, with well-developed lymphatic channels. It can also contain free nerve endings, such as Ruffini and Pacinian corpuscles, which contribute to sensory function (14, 27, 34).

There are two types of deep fascia, one is Aponeurotic fascia forms broad, sheet-like structures that attach muscles requiring a large surface area for attachment. With passage of time, tendons develops out of this type of fascia, which act as origin or insertion for other muscles i.e., aponeurotic fascia (include the fascia of the limbs), the thoracolumbar fascia, and the rectus sheath. The deep fascia is usually easily separable from the underlying muscle layer and thicker than other type of fascia. According to Gatt (2023), there are approximately 2 to 3 parallel bundles of collagen fibers in this type of fascia (14). Second is epimysial fascia, also known as epimysium, surrounds skeletal muscles and in certain situations have the potential to attach directly to the periosteum of bones. It can surround various muscle groups including the muscle of the trunk; the deep muscles of the back or the abdominal wall, pectoralis major, trapezius, deltoid, and gluteus maximus. This fascia is generally thinner compared to aponeurotic fascia and is tightly integrated with the muscle tissue via septa that penetrate the muscle layer (7).

### 2.3 Visceral fascia

Visceral fascia refers to the connective tissue located just beneath the mesothelium of the serosa and surrounding the organs (i.e., viscera). Under normal, healthy conditions, the visceral fascia is relaxed and can stretch and move freely. However, physical trauma, scarring, infection, or inflammation can impair its pliability, leading to tightness, pain, or restricted organ motion. Manual therapies often emphasize the elasticity of visceral fascia and its role in transmitting forces, as well as its potential to cause pain (35). Internal fascia is categorized into three primary functional types: the neurovascular sheaths (tunica adventitia), the visceral fasciae that directly invest internal organs, and the parietal fasciae lining the thoracic and abdominal cavities (36), The precise mapping of these fascial planes is increasingly relevant in surgical oncology and manual therapy to optimize procedural outcomes and elucidate the mechanisms of tissue mobilization (37, 38), In a physiological state, these layers act as low-friction mechanical interfaces that facilitate visceral gliding; however, injury or chronic inflammation can trigger myofibroblast activation and collagen remodeling, leading to fibrotic adhesions, tissue densification, and localized nociception. Beyond their role in visceral mobility, these tissues contribute significantly to the transmission of mechanical forces across body cavities (39). Histological analyses reveal that fascial morphology is organ-specific; for instance, the pleura, peritoneum, and pericardium exhibit distinct elastic fiber distributions tailored to the specific mechanical demands of the lungs, viscera, and heart, respectively (40, 41). Furthermore, interspecies anatomical heterogeneity is observed, such as structural variations in splenic fascia between murine and canine models (42). In the liver, the hepatic capsule (Glisson’s capsule) demonstrates a complex reticular architecture, emphasizing that fascial organization is highly specialized and lacks a uniform pattern across different organ systems (35, 43).

### 2.4 Parietal fascia

**Figure 2:**
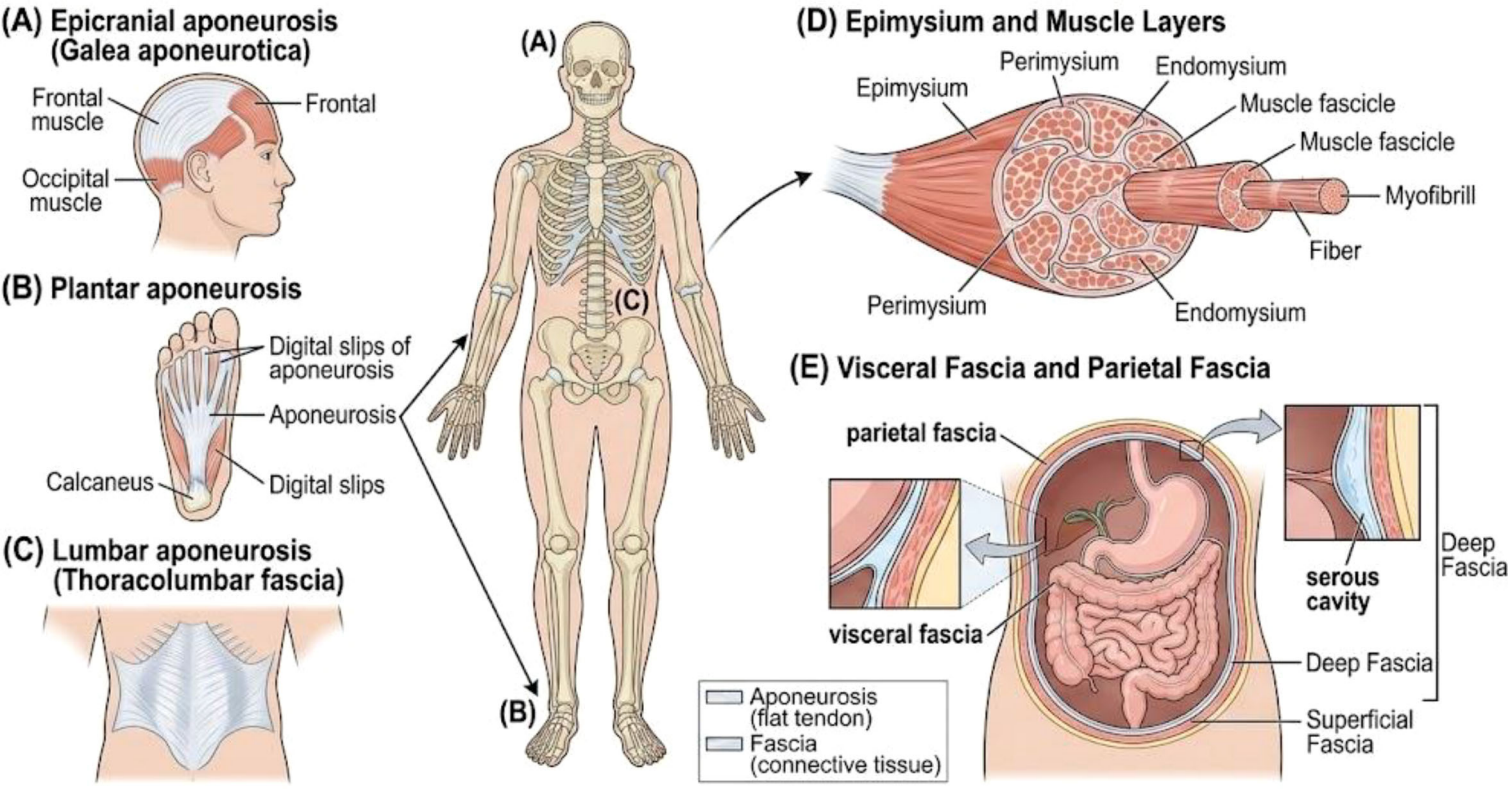
Depiction of fascia types and their anatomical locations in human body. **(A)** Epicranial aponeurotic fascia is stretched over upper head. **(B)** Plantar aponeurotic fascia stretches under feet and **(C)** Lumber aponeurotic fascia lies in the lower back. **(D)** Epimysial fascia covers the outer surface of muscles. **(E)** Visceral fascia surrounds the visceral organs and parietal fascia lines the walls of body cavities, such as the thoracic cavity (chest), abdominal cavity, and pelvic cavity.

Parietal fascia is a primary component of the internal fascia (fascia interna) system, representing the connective tissue layer that lines the internal surfaces of the body cavities. It is situated between the deep fascia of the musculoskeletal wall (such as the transversalis fascia) and the parietal layer of the serous membranes, including the pleura and peritoneum (44). Distinct from the visceral fascia, which directly envelopes the internal organs, the parietal fascia functions as a critical mechanical interface between the body wall and the viscera. This role is most prominent in the pelvic region, where the parietal component of the endopelvic fascia provides a supportive structural framework for the urogenital organs (45). By integrating with fibrous septa, the parietal fascia ensures that the viscera remain mobile relative to the musculoskeletal wall during movement and respiration, while also acting as a conduit for neurovascular structures (46). Unlike the autonomically innervated visceral fascia, the parietal fascia is characterized by somatic innervation, positioning it as the primary anatomical substrate for the ‘physical tightening’ and somatic armor frequently manifested in anxiety and panic disorders (44). Chronic stress-induced restriction of the diaphragmatic parietal fascia facilitates dysfunctional breathing patterns, which serve to perpetuate emotional dysregulation and reinforce systemic physiological distress (20). Furthermore, the parietal layers within the pelvic region often function as reservoirs for chronic tension in trauma-informed psychiatry, where fascial densification may directly distort a patient’s sense of physical self-integrity and body image (20). These aberrant peripheral signals generate ‘interoceptive noise’ that degrades the higher-order body representations and ‘grounding’ necessary for a stable psychological experience of the self (1). The details are shown in **Figure 2**.

## Function of fascia

The fascial system serves as a critical mediator of human locomotion and physiological homeostasis, demonstrating dynamic plasticity in response to environmental stimuli and functional demands. Maintaining the structural and functional integrity of this network is paramount for optimizing musculoskeletal coordination, interoceptive awareness, and the systemic modulation of hormonal, autonomic, and neurovascular processes. Collectively, these integrated fascial functions provide the biological scaffolding necessary for adaptive sensorimotor integration and purposeful interaction with the environment. The importance of fascia for physiological functions cannot be neglected which includes posture and movement (24), force transmission (47), strength generation (17), elastic recoil (48), and proprioception (24, 49). In addition to these, it also contributes to two important tasks i.e., exteroception (the sense of external stimuli) and interoception (the perception of internal bodily states) (22).

Besides these sensory functions, the fascia plays a significant role in regulating various physiological processes. These processes include protecting delicate neural and vascular structures and organs (50), enhancing lymphatic function (51), assisting in thermoregulation, and modulating inflammatory and immune responses (30). Importantly, fascia can aid in venous return (52), and also provides active support in neurotransmitter transmission, including serotonin, dopamine, GABA, and acetylcholine (53). Moreover, the fascial matrix functions as a dynamic physiological environment and a critical conduit for neurovascular signaling, serving as a mechanical interface rather than a primary secretory organ. While adipose tissue integrated within the fascial layers, specifically the superficial adipose tissue (SAT)—exhibits significant endocrine activity in the regulation of metabolic homeostasis (54), the fascial connective tissue itself acts primarily to facilitate the transport and distribution of biochemical mediators. Furthermore, the extensive sensory network of the fascia is predominantly dedicated to proprioception and interoception, providing the central nervous system with essential data regarding internal mechanical states and bodily self-awareness (21). This focus on interoceptive signaling is particularly salient in a psychiatric context, as fascial afferents inform the higher-order body representations that underpin emotional regulation and the psychological experience of the self (1). By refining this distinction, we emphasize that fascial dysfunction contributes to psychiatric symptoms through distorted internal feedback loops and interoceptive noise rather than through the perception of external environmental stimuli.

The structural development of fascia is continuous, as its development begins early in embryonic and childhood stages (55), and continues to evolve throughout life in response to functional demands. Fascia adapts continuously, and it can be affected by numerous factors such as posture, repetitive movements, the volume of motion, load (56), stress, strain, hydration (57), as well as the levels of neurotransmitters and hormones (58). Such adaptations with time enable fascia to maintain its role in supporting both the structural integrity and functional performance of the body throughout life.

## Myofascial chains

With the advancement in recent histological discoveries, the interest in studying fascia tissue and its mechanism has gained much attention. Such interest has challenged the previous assumption of considering fascia as merely a passive structure with identification of contractile cells, free nerve endings and mechanoreceptors. Instead, the prominent roles of fascia like proprioceptive and mechanically active roles have been observed in the body (59, 60). Hence, the discoveries imply that muscles do not function in isolation but are part of a dynamic, body-wide network, where fascial structures serve as linking components in a tensegrity-like system.

It has been observed that fascia has the tendency to transmit tension (61, 62), and two properties, proprioceptive and nociceptive properties, may contribute to pain that radiates to distant anatomical regions. These principles led to the concept of “myofascial meridians,” linking various body parts through muscles and fascia. Myers (2013), using cadaveric dissections, identified eleven such meridians, defined by the direct linear connection between two muscles (63). The theoretical framework for these chains was established decades earlier. Françoise Mézières first postulated the existence of posterior muscle chains in the mid-20th century, a concept later refined and expanded by Souchard into the clinical framework of Global Postural Reeducation (64). Subsequently, Luigi Stecco provided a rigorous anatomical basis for these connections by defining ‘myofascial sequences’ and ‘spirals,’ emphasizing the role of deep fascia in coordinating muscular activity across distinct anatomical segments (46). Together, these models suggest that the muscular system does not function in isolation but rather as a series of interconnected myofascial units. Under the biotensegrity model, the fascial system functions as a global tension-distributing network where mechanical restrictions in one segment are redistributed across the entire myofascial continuity (65). This systemic redistribution is a primary determinant of ‘affective posture,’ the physical manifestation of an individual’s internal emotional state. For example, chronic tension within the Superficial Back Line (SBL) can anatomically reinforce a rigid ‘defensive stance,’ a postural configuration frequently observed in patients experiencing hypervigilance or chronic anxiety (66). These body-wide continuities thus provide a structural framework for the somatization of stress, where persistent postural tone acts as a feedback loop that may sustain and exacerbate emotional dysregulation.

Despite extensive support and literature on the existence of myofascial chains, research on their functional significance, particularly in force transmission, remains limited and speculative. Further studies are required to understand the active role of these meridians in force transfer. Wilke et al. (2016) provided strong evidence for three myofascial chains described by Myers (2020), the Superficial Backline (SBL; plantar fascia, gastrocnemius, hamstrings, and erector spinae muscles), the Back Functional Line (BFL; latissimus dorsi, contralateral gluteus maximus, and vastus lateralis muscles), and the Front Functional Line (FFL; adductor longus, contralateral rectus abdominis, and pectoralis major muscles) (67, 68).

The idea existence of the Superficial Front Line (SFL) is still a topic of debate. There’s no clear structural connection between the rectus femoris and rectus abdominis muscles. On top of that, the sternalis muscle, which some believe extends from the rectus abdominis, is quite rare and doesn’t consistently connect to it either (69, 70). While the evidence for tensile transmission through myofascial pathways is compelling, much of the research has been conducted *in vitro* using cadaveric specimens. To better understand the functional relevance of myofascial chains in the movement system, randomized, controlled *in-vivo* studies are necessary (67).

## The myofascial-psychiatric interface

Myofascial Pain Syndrome (MPS) is a prevalent musculoskeletal disorder characterized by regional pain originating from myofascial trigger points (MTrPs)—exquisitely tender loci situated within palpable taut bands of skeletal muscle or their associated fascia (71–73). Clinically, MPS manifests as a complex of sensory, motor, and autonomic symptoms, including restricted range of motion (ROM), localized muscular weakness, and referred pain patterns (74). Diagnosis remains fundamentally clinical, predicated on a systematic palpatory examination and a detailed review of patient history to differentiate its multifaceted presentation from other pain-related syndromes (75, 76).

Pathologically, MPS is categorized as primary when it occurs in isolation, or secondary when it arises in conjunction with comorbid medical conditions. The etiology of the MTrP is traditionally attributed to sustained contractile activity, which precipitates a localized metabolic ‘energy crisis’ characterized by ischemia and hypoxia. This hypoxic environment triggers a cascade of neurophysiological alterations in nociceptors, facilitating peripheral sensitization and the expansion of pain (50). Recent advancements have expanded this muscular focus to include the fascial matrix, suggesting that alterations in the extracellular matrix specifically fascial densification play a critical role in the persistence of nociceptive signaling (77, 78).

From an epidemiological perspective, myofascial pain affects an estimated 85% of the general population at least once during the lifespan (79). Among clinical populations presenting with musculoskeletal complaints, the incidence of MPS ranges from 30% to 93%, with nearly half of these patients exhibiting active MTrPs upon physical examination (80). Given its significant prevalence and multifactorial nature, MPS represents a critical component in the differential diagnosis of chronic pain and requires an integrated therapeutic approach targeting the entire myofascial complex (77).

The intersection of fascial integrity and psychiatric health is characterized by a bidirectional relationship between structural densification and neurological processing (81). Myofascial trigger points, frequently comorbid with anxiety and depressive disorders, are increasingly understood as products of fascial densification, specifically the increased viscosity of hyaluronan, which impairs tissue gliding and maintains a pro-nociceptive environment (79) (81). This persistent fascial nociception can trigger central sensitization, a state of neuronal hyper-excitability that reorganizes emotional and affective processing within the amygdala and the anterior cingulate cortex (82). Consequently, the slouched posture and altered gait patterns observed in depressed individuals are not merely symptomatic markers, but components of a maladaptive feedback loop of embodiment; in this cycle, a stiffened fascial system generates ‘proprioceptive noise’ that actively reinforces depressive cognitions and degrades the psychological experience of the self (1, 83).

## Risk factors for development of myofascial pain

Risk factors including age, sex, occupational factors, and psychological factors contribute to myofascial pain. Aging has been found to have a direct association with higher prevalence of myofascial trigger points, possibly due to age-related changes in muscle structure and function. Over the years, muscles may become less flexible and more prone to developing areas of hypercontracted muscle fibers, leading to the formation of trigger points. Sex also plays a significant role, with women being more likely to develop myofascial pain than men, perhaps related to hormonal fluctuations and inherent differences in muscle physiology between the sexes (84).

Moreover, the influence of psychological factors such as stress, anxiety, and depression may also contribute to the onset of myofascial pain. Recent meta-analytical evidence highlights a divergence in pain perception patterns among individuals with mood disorders; while anxiety is characterized by a generalized reduction in pain thresholds, depression is associated with modality-specific responses, typically exhibiting higher thresholds for external stimuli but lower thresholds, and thus increased sensitivity, for interoceptive stimuli (85), and trigger points may form due to chronic stress, causing sustained muscle tension. Not surprisingly, myofascial pain often coexists with comorbid conditions, including spinal pathologies, fibromyalgia, temporomandibular disorders (TMD), and chronic tension-type headaches (CTTH), complicating diagnosis and treatment. Therefore, understanding the interplay between myofascial pain and these co-occurring disorders is crucial for effective management and improving patient outcomes. Furthermore, future research is essential to uncover the underlying mechanisms and to develop more effective strategies for addressing these complex and often overlapping pain syndromes (86).

## Mental health and myofascial pain

Systematic evidence underscores a robust bidirectional nexus between physical and mental health, reporting that roughly 40% of individuals with chronic pain suffer from clinically significant anxiety and depression (87).Patients seeking care for musculoskeletal pain who also experience anxiety and depression tend to report greater physical limitations and more significant pain interference, affecting aspects of their lives such as social, cognitive, emotional, physical, and recreational (88–90). While treating mental health conditions such as anxiety and depression has been shown to improve physical function and reduce pain-related limitations, the reverse, addressing physical pain directly may also lead to improvements in mental health symptoms (91–93).

While many clinicians and patients focus on addressing physical health issues, assuming that mental health symptoms will improve as physical conditions improve (94, 95). This approach may be influenced by barriers to accessing mental health care, such as societal stigma, financial constraints, and a global shortage of mental health professionals (96, 97). Additionally, the structure of medical training often means that healthcare providers who specialize in physical impairments are not routinely trained to address the mental health aspects of physical pain and dysfunction (98, 99).

## Myofascial pain and depression

Major depression occurs in 30% to 54% of chronic pain patients (100), and a likely relationship exits between myofascial pain and depression. When comparing depression and somatization between individuals with myofascial pain syndrome (MPS) and those with other etiologies of pain, such as disc displacements, higher levels of depression and somatization were observed in individuals with myofascial pain (101). Other findings revealed a link between depression, anxiety, and pain intensity in patients with myofascial trigger points (MTrPs), such as in the upper trapezius (102). Physical symptoms of depression, such as muscle tension, back and limb aches, and muscle fatigue, are often exacerbated by chronic pain, creating a cycle in which emotional distress from depression triggers muscle tension and promotes the formation and persistence of myofascial trigger points (103). Depression not only amplifies an individual’s sensitivity to pain but also heightens their awareness of physical discomfort, further increasing muscle tension and contributing to myofascial pain (77).

Research suggests that emotional stressors, such as trauma or loss, can trigger a series of physiological reactions, including increased muscle tension (104). Prolonged muscle contraction may contribute to the development of myofascial trigger points (MTrPs), thereby perpetuating a cycle of pain and emotional distress (104). A research reported that many individuals with depression reported physical symptoms as their primary reason for seeking medical care, highlighting the significant impact of chronic pain on depressive symptoms (104). Similarly, Okumus et al. (2010), identified a positive correlation between myofascial pain syndrome and depression, noting that higher pain intensity corresponded with more severe depressive symptoms. This study highlighted that greater pain severity was linked to an increased risk of depression (105). Additionally, Kroenke et al. (1994) reported that the severity of depression closely aligned with the number of physical symptoms patients experienced, with 60% of depressed individuals reporting nine or more symptoms. These findings illustrate the complex interaction between myofascial pain and depression, where each condition exacerbates the other (106).

Beyond clinical symptoms, depressed individuals exhibit distinct biomechanical alterations in their myofascial tissue, specifically increased stiffness and decreased elasticity. These changes are increasingly understood through recent histological studies have demonstrated a high density of sympathetic fibers within both the superficial and deep fasciae, primarily associated with the control of blood vessels (107). As the importance of fascial tissue cannot be ignored for force generation and proprioception, still the prolonged dysfunction can result in chronic body tension and impaired motor control (108). In states of chronic psychological stress or depression, persistent sympathetic overactivity may induce localized vasoconstriction within the fascial layers. This subsequent ischemia creates a hypoxic environment that promotes the activation of myofibroblasts and the excessive deposition of extracellular matrix components (109). Over time, this ischemic state can evolve into tissue fibrosis and the densification of hyaluronan, which further compromises tissue gliding and increases mechanical stiffness (81). Such findings may be helpful in explaining why depression is commonly linked to altered gait patterns, such as diminished arm swing and slouched posture (110, 111). These physical expressions of depression can further intensify negative emotions and thoughts, perpetuating a cycle of physical and psychological distress (112). It is important to recognize the relationship between myofascial pain and depression, as addressing one aspect of the condition may provide relief for the other, and can allow for improved diagnosis and treatment.

## Body stiffness in depression and fascia

Fascial stiffness and elasticity are modulated over varying time scales, from minutes to months, through biochemical and biomechanical mechanisms. The contractile behavior of fascial cells is influenced by cytokines present in the extracellular matrix’s ground substance. Notably, the cytokine TGF-β1, which plays a role in fascial regulation, is affected by autonomic nervous system activity (113). Since major depressive disorder (MDD) is associated with autonomic nervous system dysregulation and immune dysfunction including increased TGF-β1 levels (114, 115). This biochemical environment triggers the differentiation of fibroblasts into contractile myofibroblasts, which express alpha-smooth muscle actin (α-SMA) and generate a persistent, non-muscular contractile tone within the fascial matrix (109). Unlike transient muscular recruitment, this myofibroblast-mediated contraction results in long-term structural stiffening and reduced elasticity (109), providing a functional explanation for the somatic heaviness and restricted mobility frequently reported by patients (116). Furthermore, this stiffened fascial architecture may act as a biological reservoir for stress, continuously generating aberrant interoceptive signals that reinforce the brain’s higher-order representations of a ‘defensive self,’ thereby sustaining the cycle of affective dysregulation (1).

## The psychophysiology of fascial hypervigilance

Chronic pain syndromes, specifically Myofascial Pain Syndrome (MPS), exhibit significant comorbidity with, including generalized anxiety disorder and post-traumatic stress disorder (PTSD) (117–119). Clinical evidence indicates that patients with regional myofascial pain report more frequent and severe anxiety symptoms compared to healthy cohorts, with the severity of anxiety independently correlating with the prevalence of myogenic tender points (120–122). High anxiety has been reported in individuals with myogenic fascial pain, with the severity of anxiety independently correlating with the presence of muscle tender points across all study groups. Similarly, Maslak-Beres et al. (2019) reported elevated stress levels in patients experiencing temporomandibular pain, irrespective of its underlying cause. Thus, such findings showed bidirectional relationship between anxiety and myofascial pain, intensifying each other, creating a reinforcing cycle of distress (112). Similarly, investigating 35 young students over an academic year showed heightened anxiety levels and increased muscle tenderness during examination periods, even among those without a prior history of pain (123). Such findings revealed that stress, anxiety, and emotional distress may not only increase pain perception but also play a role in the development of myofascial pain.

The fascial system’s role as a biological record of psychological trauma is further evidenced in trauma-exposed populations. In war veterans diagnosed with PTSD and comorbid depression, the prevalence of upper-body myofascial pain reaches 58%, a rate significantly exceeding general population averages (124). This state of ‘fascial hypervigilance’ is maintained by chronic sympathetic hyperarousal, which triggers the TGF-β1-induced differentiation of myofibroblasts, resulting in a persistent, non-muscular contractile tone (109). This structural ‘stiffening’ of the fascial matrix degrades interoceptive clarity, leading to interoceptive failure; when peripheral signals are distorted by tissue densification, the brain receives ‘interoceptive noise’ rather than clear physiological data. This mechanism provides an anatomical explanation for the emotional blindness and somatosensory amplification characteristic of alexithymia, where the individual is unable to accurately identify or regulate emotional states due to a compromised bodily feedback loop (1).

This peripheral-central interplay is anchored by neurophysiological evidence from Transcranial Magnetic Stimulation (TMS) and Quantitative Sensory Testing (QST). In patients with myofascial pain, anxiety correlates significantly with shortened cortical silent periods and increased intracortical facilitation, indicating impaired inhibitory control and central hyperexcitability (125). These findings suggest that cognitive patterns such as catastrophizing or suppressed anger are not merely psychological events but are translated into measurable cortical excitability. Consequently, the fascial system and the motor cortex form a bidirectional circuit where chronic myofascial nociception reorganizes emotional processing in the amygdala and cingulate cortex, effectively anchoring the patient in a persistent state of physiological and psychological distress (126, 127).

The reciprocal relationship between sleep architecture disturbances and chronic pain is well-documented, with sleep disorders reported in 67–88% of chronic pain cohorts (128). Conversely, approximately 50% of individuals diagnosed with clinical insomnia concurrently present with chronic pain (129), suggesting a bidirectional vulnerability where sleep disturbances frequently precede the development of both localized and widespread musculoskeletal pain (130, 131).

While specific literature regarding Myofascial Pain Syndrome (MPS) remains emerging, a comprehensive 10-year longitudinal analysis of the Taiwan National Health Insurance Research Database (n=7,895) demonstrated that individuals with insomnia exhibit a two fold increased risk of developing MPS compared to controls (132–135). These findings suggest that impaired sleep may serve as a critical neurobiological risk factor that lowers the pain threshold and facilitates the onset of myofascial trigger points.

The convergence of chronic stress, anger suppression, and sleep disturbances culminates in a state of ‘fascial hypervigilance,’ where the fascial matrix serves as a biological record of psychological trauma. This state is characterized by myofibroblast-mediated fascial stiffening and densification, which significantly compromises the tissue’s role as our primary interoceptive substrate (136). In this framework, the high prevalence of Alexithymia among myofascial pain patients reflects a functional Interoceptive Failure; as the fascia becomes increasingly rigid and densified, the interoceptive afferents reaching the brain become distorted or attenuated (137). This resulting ‘interoceptive noise’ prevents the central nervous system from accurately identifying and regulating internal emotional states, providing a tangible neurobiological basis for the emotional blunting and somatosensory amplification seen in these populations (1). Thus, maladaptive emotional regulation strategy such as suppressed anger are somatized into the fascial architecture, creating a feedback loop where peripheral structural changes directly degrade higher-order body representations and the psychological experience of the self (127, 138).

## Discussion

The synthesized evidence indicates that the fascial network functions not as a passive scaffold, but as the primary architectural substrate of the bodily ‘Self.’ Within the interoceptive framework (1), fascial integrity is a fundamental prerequisite for the brain’s construction of precise body representations. As a tissue richly innervated with sensory neurons, the fascial matrix utilizes continuous mechanotransduction, the process by which mechanical stimuli are converted into biochemical signals, to supply the central nervous system (CNS) with the high-fidelity interoceptive signaling necessary to maintain a coherent internal model (139). Conversely, chronic stress and trauma generate “interoceptive noise” by inducing fascial stiffness and adhesions, which disrupt this bidirectional communication and anchor the individual in a state of persistent psychological dysregulation (139). For instance, fascial tension in the thoracolumbar region has been associated with heightened sympathetic nervous system activity, a hallmark of anxiety disorders, suggesting that fascial health is a critical determinant of emotional and psychological resilience (116).

Beyond its role in neural signaling, fascial connective tissue acts as a critical medium for metabolic, endocrine, and immune exchanges, facilitating the return of water, proteins, and immune cells to the bloodstream through the lymphatic system. However, chronic stress, a well-established risk factor for psychiatric conditions, can cause fascial inflammation and matrix compaction (140). This stiffness may, in turn, exacerbate stress by impairing the body’s ability to relax and recover, creating a vicious cycle where fascial restrictions impinge upon the vagus nerve (116). Dysfunction in this parasympathetic regulator has been implicated in disorders such as depression and anxiety, further evidenced by the high comorbidity between psychiatric disorders and myofascial pain syndrome (141). Because pain and mental health are closely intertwined, the fascia’s role in pain perception suggests that addressing fascial health could have profound therapeutic implications for psychiatric recovery (142, 143).

**Figure 3:**
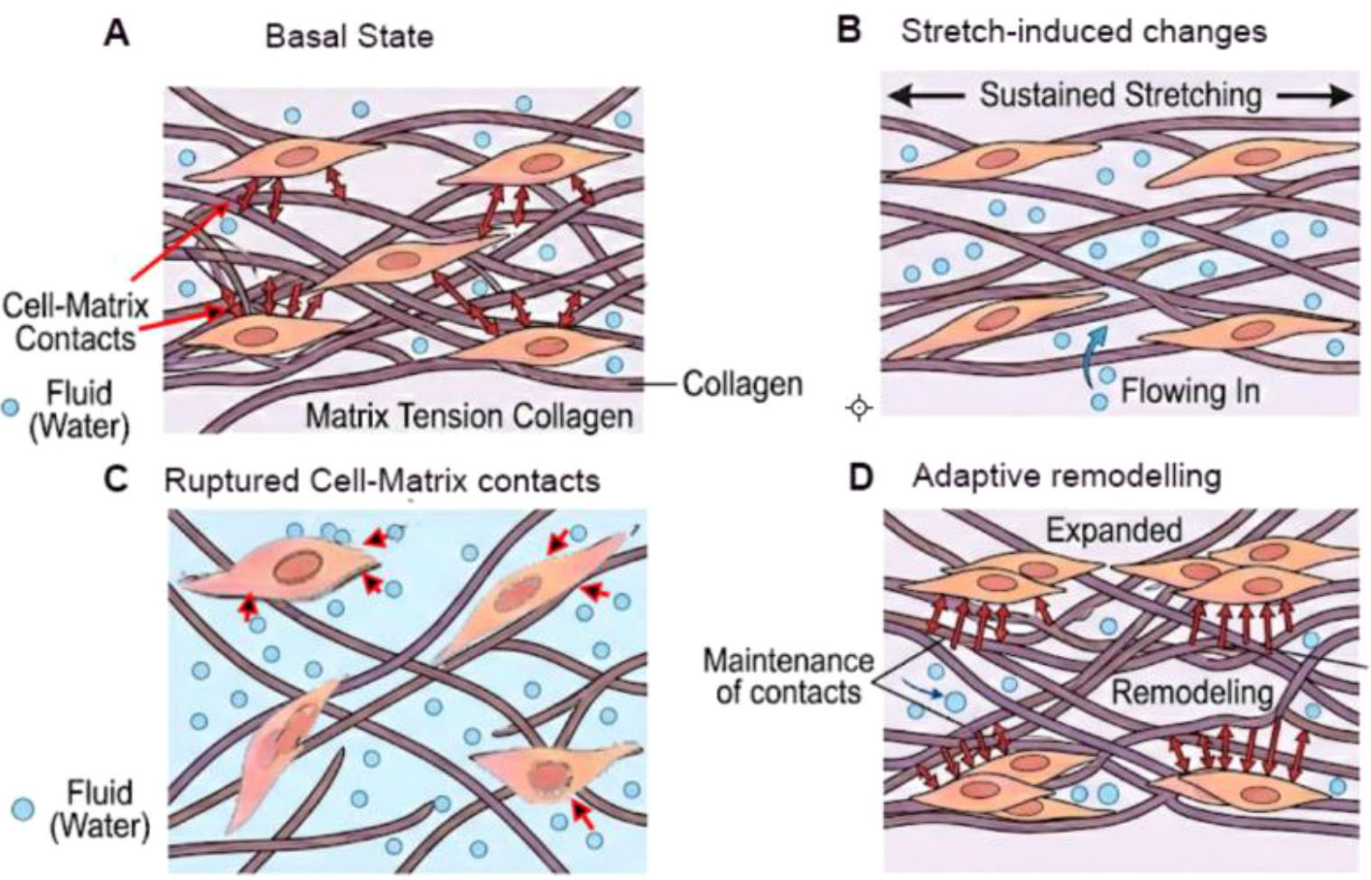
Proposed mechanism for fibroblast control of matrix tension and fluid flux in response to tissue stretch. **(A)** Fibroblasts maintain tension on the extracellular matrix and prevent fluid influx into the tissue. **(B)** Sustained stretching of the matrix for several minutes decreases matrix compaction and increase in pore size, allowing water to flow in. **(C)** Fibroblasts “letting go” of the cell-matrix contacts would further unrestrain the matrix and cause further swelling, similar to what happens during inflammation. **(D)** Fibroblast remodeling, expansion and maintenance of cell-matrix contacts would keep the matrix restrained and reduces water influx into the tissue Langevin et al. (116).

In this light, interventions such as Myofascial Release, Yoga, and Meditation serve as critical agents for fascial remodeling and the restoration of vagal tone. Prolonged stretching over several minutes has been shown to reduce matrix compaction and increase pore size within the network, facilitating the influx of water and enhancing tissue hydration (116) (**Figure 3**). By breaking the bio-behavioral freeze, a maladaptive loop of fascial densification and sympathetic hyperarousal, these modalities facilitate hyaluronan rehydration and myofibroblast modulation (109). This process resolves the physiological ‘imprint’ of trauma and restores essential tissue gliding, effectively shifting the organism from a state of defensive hyperarousal toward somatic and psychological homeostasis (109).

**Figure 4:**
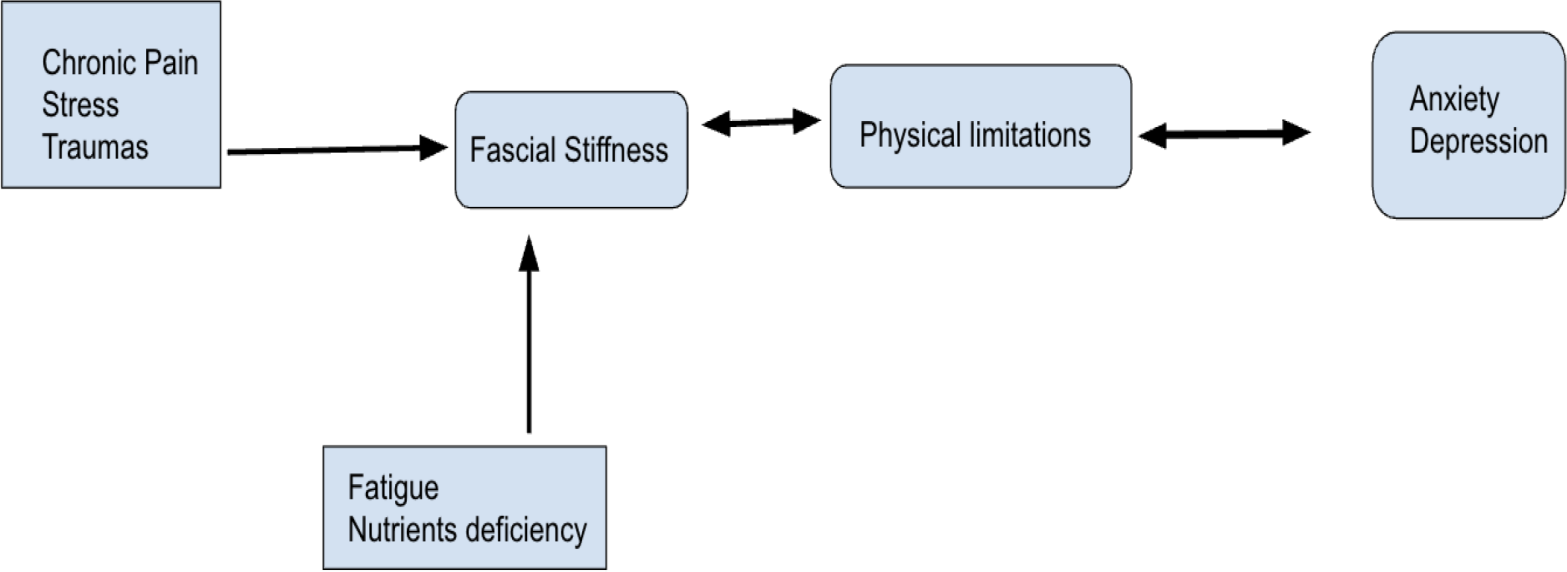
Intermediation of myofascial pain and psychiatric disorder. Created by Shah SM.

Emerging therapies such as myofascial release and manual fascial therapies have shown promise in alleviating symptoms of both physical and mental health conditions. These techniques aim to restore fascial flexibility and reduce tension, potentially improving nervous system function and emotional well-being (141). Yoga, a form of exercise that emphasizes stretching and physical engagement, has the potential to significantly alleviate fascial stiffness and contribute to mood regulation. The practice of yoga promotes flexibility, enhances blood flow, and encourages the release of tension within the fascial network, which may help reduce stiffness and improve overall physical function (144). Additionally, the mindful and rhythmic nature of yoga can positively influence emotional well-being, aiding in mood stabilization (145). In parallel, meditation serves as a powerful tool for fostering self-driven calmness and mental clarity. By reducing hyperactivity in the brain, particularly in individuals with neurological or stress-related conditions, meditation may facilitate homeostatic restoration and modulation of parasympathetic tone, providing a physiological basis for the stabilization of emotional states (146). Together, yoga and meditation offer a holistic approach to addressing both physical and mental health challenges, making them valuable complementary practices in managing fascial stiffness and enhancing psychological resilience. Although establishing causal relationships between fascia and mental health presents significant experimental challenges, further research is essential to elucidate these connections. Emerging evidence suggests that fascia may play a pivotal role in the mind-body continuum, potentially serving as a novel target for integrative treatments in psychiatry. By exploring the interplay between fascial health and mental well-being, it may be possible to develop therapeutic interventions that reduce the reliance on psychiatric medications. The details are shown in **Figure 4**.

## Conclusion

In conclusion, the fascia’s role in the body extends far beyond structural support, potentially influencing mental health through mechanisms such as stress response modulation, pain perception, and neural communication. This framework proposes a shift toward an embodied psychiatric practice, where the fascia is understood as an integrated system that archives the somatic impact of emotional distress. Incorporating these body-based therapies offers a powerful synergy with traditional pharmacological treatments, providing a more holistic and comprehensive strategy for long-term mental health recovery. Future studies should focus on elucidating the specific pathways through which fascia interacts with the brain and exploring the therapeutic potential of fascial interventions. Moreover, Yoga and meditation could be considered for future research as integrative approaches to alleviate the symptoms of psychiatric diseases.
